# Targeting ferroptosis in acute kidney injury

**DOI:** 10.1038/s41419-022-04628-9

**Published:** 2022-02-24

**Authors:** Lihua Ni, Cheng Yuan, Xiaoyan Wu

**Affiliations:** 1 Department of Nephrology, Zhongnan Hospital of Wuhan University, Wuhan, 430071 China; 2 Department of Gynecological Oncology, Zhongnan Hospital of Wuhan University, Wuhan, 430071 China

**Keywords:** Acute kidney injury, Cell death

## Abstract

Acute kidney injury (AKI) is a major public health problem with high incidence and mortality. As a form of programmed cell death (PCD), ferroptosis could be considered as a process of iron accumulation and enhanced lipid peroxidation. Recently, the fundamental roles of ferroptosis in AKI have attracted much attention. The network mechanism of ferroptosis in AKI and its roles in the AKI to chronic kidney disease (CKD) transition is complicated and multifactorial. Strategies targeting ferroptosis show great potential. Here, we review the research progress on ferroptosis and its participation in AKI. We hope that this work will provide clues for further studies of ferroptosis in AKI.

## Facts

- Ferroptosis is an iron- and reactive oxygen species (ROS)-dependent lipid peroxidation that is different from the other forms of programmed cell death (PCD) at the morphological and biochemical levels.
- Ferroptosis participates in the occurrence and development of acute kidney injury (AKI).
- Numerous studies have demonstrated that strategies targeting ferroptosis can delay the progression of AKI.

## Open questions

- How can ferroptosis be detected?
- What is the interplay of mitochondria and lysosomes, the ER, and the Golgi in ferroptosis?
- How does Golgi stress induce ferroptosis?
- How does the complicated network mechanism regulate ferroptosis?
- What is the role of ferroptosis in the AKI to chronic kidney disease (CKD) progression?
- What is the interplay of ferroptosis and necroptosis in the progression of AKI?

## Introduction of acute kidney injury (AKI)

The prevalence of acute kidney injury (AKI) is increasing in hospitalized patients [1–3]. The incidence ranges from 10–15% in all hospitalizations and is as high as 50% in the intensive care unit (ICU) [4, 5]. In addition, it demonstrated that AKI increase the potential risks of chronic kidney disease (CKD), cardiovascular disease, and end-stage renal disease (ESRD) [6–8]. Generally, several factors, such as ischemia, insufficiency of the circulating blood volume, nephrotoxic drugs, and urinary tract obstruction, lead to AKI. Apart from blood purification, there are few effective treatments for AKI. Untreated AKI would continue to cause further damage and is associated with a poor prognosis. Thus, exploring novel targets or drugs is desperately needed.

The pathogenesis of AKI is considered to involve nephrotoxicity, the inflammatory response, acute tubular hypoxia and necrosis, pericyte injury, and microvascular injury/dysfunction [9–13]. Recently, ferroptosis has been demonstrated to be involved in the pathogenesis and therapeutic strategies of AKI. Ferroptosis induced a first wave of death, triggering an inflammatory response that in turn contributed to the deterioration of renal function. And strategies targeting ferroptosis showed great therapeutic potential. Our study summarizes the current progress on ferroptosis and its roles in AKI.

## Ferroptosis and cell death

### Ferroptosis (one center, two characteristics, and four aspects)

The concept of ferroptosis was first proposed in 2012 by Dixon et al. [14]. The characteristics of ferroptosis have been studied for several years and can be summarized as one center, two characteristics, and four aspects [15–17]. One center refers to the accumulation of lipid reactive oxygen species (ROS). Two characteristics refer to the destruction of cellular antioxidants and enhanced levels of intracellular iron, which lead to the deposition of detrimental lipid ROS. Four aspects indicate metabolic disorder of iron, the amplified production of ROS and accumulation of lipid peroxide, and the consumption of glutathione peroxidase 4 (GPX4) and system Xc^−^ (a cysteine/glutamate antiporter system).

Morphologically, ferroptosis mainly manifests as shrinkage of mitochondria with the rupture of mitochondria membrane, enhanced membrane density, and reduction or disappearance of the mitochondrial crest [18]. Based on the morphological particularity of ferroptosis, transmission electron microscopy (TEM) is widely applied to observe ultrastructural changes [19].

Biochemically, ferroptosis mainly includes the consumption of glutathione (GSH) and the decreased activity of GPX4. In addition, the metabolism of iron, amino acids, and ROS is also associated with ferroptosis [20, 21].

### Comparisons of the common types of cell death

Cell death can be classified into programmed cell death (PCD, such as ferroptosis, apoptosis, necroptosis, and autophagy-dependent cell death) and accidental cell death (such as necrosis) [22–25]. As an iron-dependent nonapoptotic cell death, ferroptosis is different from other cellular deaths (apoptosis, necrosis, and autophagy) [26–29]. The accumulation of free iron is a key initiator of ferroptosis. During ferroptosis, the cell membrane is destabilized, the cytoskeleton is rearranged, and proteostasis is disrupted. Green et al. suggested that ferroptosis could be best described as a cellular “sabotage”, meaning that normal metabolic functions lead to cell death [29].

Apoptosis is the most common type of PCD. During apoptosis, cells shrink, the plasma membrane bubbles, and apoptotic bodies form. Abundant cellular proteases contribute to the disintegration of skeletons, membranes, and proteins [30–32]. We also consider apoptosis to be a process of self-killing.

Autophagy-dependent cell death is a natural degradation process of cellular contents and a rare kind of PCD [33–35]. During autophagy-dependent cell death, extensive vacuolization of the cytoplasm is formed; the integrity of the plasma membrane is lost; and sometimes, enlargement of the Golgi and ER is observed [36–38]. We also consider autophagy-dependent cell death to be a process of self-eating.

Necroptosis, a widely studied programmed necrosis, is mediated by the receptor-interacting protein kinase 1 (RIPK1), receptor-interacting protein kinase 3 (RIPK3), and mixed lineage kinase domain-like protein (MLKL). During necroptosis, the cytoplasm was swelling, the plasma membrane was raptured and the intracellular content was spilling.

Necrosis is a kind of unregulated cell death that comes from stress stimuli. During necrosis, the cell volume is increased, the integrity of the plasma membrane is damaged, and the cellular contents are lost [39–42]. Decay products recruit lymphocytes, leukocytes, and macrophages to phagocytose dead cells.

**Fig. 1:**
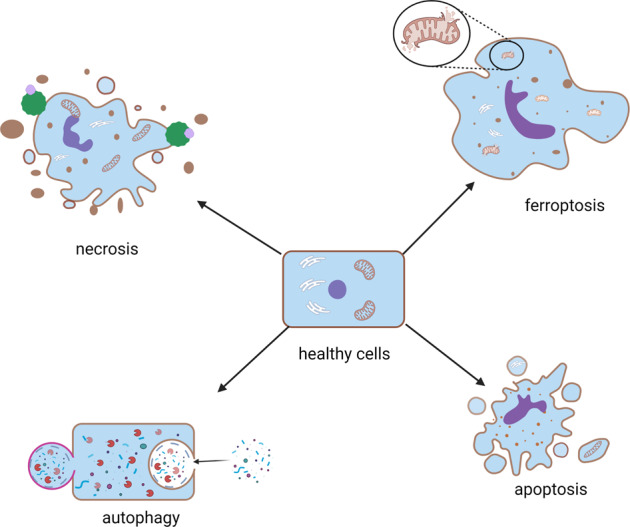
Schematic representation of the main types of cell death. Cell death can be classified into programmed cell death (PCD, such as ferroptosis, apoptosis, and autophagy) and accidental cell death (such as necrosis).

Above all, there are many differences among the various kinds of cell death (Fig. 1). Ferroptosis is different from the others in morphology and biochemistry. Iron-dependent lipid peroxidation is the most important feature indicating that ferroptosis is different from the other kinds of cell death.

## The detection of ferroptosis

It is worth noting that to date, there is no specific method for the detection of ferroptosis. According to the features of ferroptosis, the most frequent types of detection can be divided into five parts: cell viability, cytotoxicity and death, levels of iron, ROS, and biomarkers and morphologic changes (Table 1). A cell counting kit-8 (CCK-8) assay can be applied to detect cell viability. Assay of lactate dehydrogenase (LDH) release was used to analyze cell cytotoxicity. Ferroptotic cellular death can be assessed by TUNEL assay. The detection of serum nonheme iron can be conducted using an iron/TIBC reagent. The chromogen method can detect tissue nonheme iron. Malondialdehyde (MDA) and 4-hydroxynonenal (4HNE) can be used to evaluate iron-related lipid peroxidation. In addition, the levels of GSH and GPx4 can be measured to study the inhibition of antioxidants during ferroptosis. Detecting ferroptosis-associated biomarker proteins, such as nuclear respiratory factor 2 (NRF2), nuclear receptor coactivator 4 (NCOA4), heme oxygenase-1 (HO-1), GPX4, and heavy peptide ferritin (FTH), are conventional methods. As mentioned above, TEM can also be used to observe the ultrastructural changes during ferroptosis.

**Table 1:** The measurement of ferroptosis.

Detection	Methods	References
Cell viability, cytotoxicity, and death	CCK-8, LDH release, and TUNEL	[136, 137]
Iron levels	Flow cytometry or confocal via Phen Green SK probes, chromogen method, Iron/TIBC reagent	[138]
ROS levels and lipid peroxidation	MDA, 4HNE, flow cytometry via BODIPY and DCFH-DA, JC-1, GSH	[139–142]
Mitochondrial oxidative stress	Mito TEMPO	[143]
Biomarker proteins	NRF2, NCOA4, HO-1, GPX4, FTH, COX2, PTGS2, ACSL4, NOX1, SLC7A11, metallothionein-1	[126]
Morphology	TEM	[144, 145]

Note: CCK-8 Cell Counting Kit, NRF2 nuclear respiratory factor 2, NCOA4 nuclear receptor coactivator 4, HO-1 heme oxygenase-1, FTH heavy peptide ferritin, TEM transmission electron microscopy, ACSL4 acyl-CoA synthetase long-chain family member 4, NOX1 NADPH oxidase activator 1, SLC7A11 cysteine/glutamate antiporter solute carrier family 7 member 11.

Generally, it is encouraged to perform five or more levels of methods together—in other words, the greater the better.

## Regulatory mechanism of ferroptosis

**Fig. 2:**
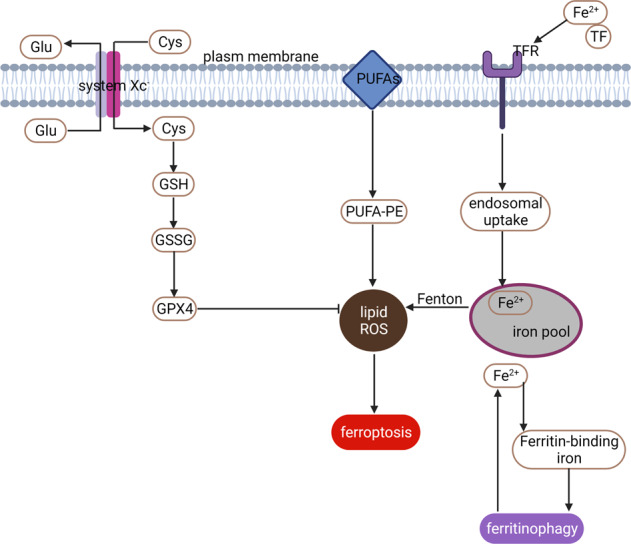
Regulatory mechanism of ferroptosis. General mechanism of ferroptosis associated with System Xc−, GPX4, iron homeostasis, ROS, and lipid peroxidation.

The mechanism of ferroptosis has been widely studied. System Xc^−^, GPX4, iron homeostasis, ROS, and lipid peroxidation are the most extensively studied mechanisms (Fig. 2).

### System Xc^−^

As cysteine/glutamate reverse transporters expressed on the plasma membrane, system Xc^−^ donates intracellular glutamate and takes extracellular cystine at a molar ratio of 1:1 [43]. System Xc^−^ includes two subunits, light chain xCT, and heavy chain 4F2hc. Inhibition of system Xc^−^ contributes to decreased expression of GSH and different types of cell death. In addition, xCT is a major gene for the regulation of iron overload/ferroptosis via gene chip screening. Further studies have indicated that xCT attenuates ferroptosis during iron overload [44, 45]. Some agents, such as sorafenib, sulfasalazine, and glutamate, can trigger ferroptosis by blocking system Xc^−^ [46].

Briefly, impairment of the system Xc^−^ dependent antioxidant defense system contributes to oxidative damage and thus triggers ferroptosis. Erastin induces ferroptosis by blocking system Xc^−^ [46]. However, due to the lack of specificity and off-target effects, the development of therapies targeting xCT has been constrained [47]. Hence, finding a highly effective and specific xCT blocker is of vital importance.

### GPX4

GPX4 is a key enzyme that facilitates GSH to counteract lipoxygenase (Alox) activity and phospholipid/cardiolipin oxidative events [48]. RSL3 binds and further inactivates GPX4 to induce ferroptosis by decreasing the antioxidant capacity and the accumulation of ROS [49]. GPX4 knockout in tubular cells leads to ferroptosis and massive cell death [50]. Yang et al. demonstrated that cells with downregulated expression of GPX4 were more sensitive to ferroptosis, while upregulated GPX4 inhibited ferroptosis [51]. In response to GPX4 inactivation and system Xc^−^ inhibition, cells undergoing ferroptosis participate in the iron-dependent decrease in polyunsaturated fatty acids (PUFAs) and the accumulation of L-ROS [52, 53]. PUFAs contribute to the formation of lipid hydroperoxides (L-OOH). Iron and PUFAs give rise to enhanced toxic levels of L-ROS. Friedman Angeli et al. had proved the involvement of GPX4 activation in AKI [50]. And irisin could attenuate IRI-induced AKI through upregulating GPX4 [54]. The recent work showed that IRI-induced renal ferroptosis is regulated by an augmenter of liver regeneration (ALR), which is associated with the GSH-GPX4 system [55].

Simply put, RSL3 induces ferroptosis by directly inhibiting GPX4. Inactivated GPX4 leads to enhanced production of lipid-based ROS and lipid hydroperoxides, which ultimately induce ferroptosis [16].

### Iron homeostasis

Ferroptosis is characterized by iron-dependent oxidative damage to some degree [15, 56]. Excessive iron causes harm by generating ROS and regulating lipid peroxidation.

Physiologically, internal absorption- and erythrocyte degradation-derived Fe^2+^ can be oxidized to Fe^3+^. Fe^3+^ can enter the cells by bonding to transferrin (TF), which forms the complex of “TF–Fe^3+^–TFR1” by TF receptor 1 (TFR1). Then, the intracellular Fe^3+^ is further converted to Fe^2+^ and stored in the iron pool [57, 58].

Pathologically, excess Fe^2+^ can be oxidized to Fe^3+^ and exported by ferroportin (FPN), which increases the production of ROS through the Fenton reaction and ultimately induces ferroptosis. Ferritin is a main intracellular iron storage protein. Ferritin can be divided into ferritin light chain (FTL) and ferritin heavy chain (FTH). The FTL facilitates the iron nuclei formation, while the FTH promotes ferrous oxidase. Collectively, ferritin serves to iron storage and utilization. Inhibiting TFR1 can alleviate erastin-induced ferroptosis [59, 60]. Blocking iron response element-binding protein 2 (IREB2), the main transcription factor for iron hemostasis, can obviously alleviate erastin-induced expression of FTL and FTH. The role of ferritin in preventing sepsis-induced AKI had been aroused. More research about the functions of ferritins in sepsis-induced AKI might be encouraged [61].

Taken together, the disturbance of iron metabolism in favor of iron overload contributes to ferroptosis.

### ROS and lipid peroxidation

Enhanced accumulation of ROS is one of the major causes of ferroptosis. Erastin treatment results in inhibiting system Xc^−^, which further initiates mitochondrial dysfunction, disturbances of cellular redox homeostasis, and the formation of lipid peroxides. All these combined actions motivate ferroptosis [17, 62].

Erastin increases the production of lipid ROS by inhibiting system Xc^−^, which ultimately induces ferroptosis [63]. RSL3 enhances lipid peroxidation by directly binding to GPX4, which ultimately triggers ferroptosis [53, 64]. Additionally, numerous lipophilic antioxidants, such as ferrostatin-1 (Fer-1), liproxstatin-1, β-carotene, and a-tocopherol, have been proven to suppress ferroptosis [65].

In summary, lipid metabolism is significantly associated with ferroptosis.

### Others

As a proinflammatory form of PCD, ferroptosis can be regulated by several small molecules and genetic manipulation. Recent publications are summarized in Table 2.

**Table 2:** Mediators or modulators of ferroptosis.

Proteins	Name	Mechanism	References
VDACs	voltage-dependent anion channels	Production of mitochondrial ROS and mitochondrial dysfunction	[146]
p53	cellular tumor antigen p53	System Xc^−^ inhibition	[147, 148]
FSP1	ferroptosis suppressor protein 1 (a CoQ oxidoreductase, which)	FSP1 can reduce phospholipid	[149]
Panx 1	Pannexin 1	Panx 1 negatively regulates lipid peroxidation through the MAPK pathway	[126]
NRF2	nuclear respiratory Factor 2	NRF2 can bind to ARE elements in the promoter regions of the target genes	[150]
CoQ	coenzyme Q (a subtract of the oxidoreductase)	The CoQ oxidoreductase FSP1 acts parallel to GPX4 to inhibit ferroptosis	[151]
HSPB1	heat shock 27 kDa protein 1	HSPB1 phosphorylation is negatively regulated and iron-mediated in the production of lipid ROS	[152]
VDR	Vitamin D receptor	VDR mediates the transcription of GPX4	[73]
NOX	NADPH oxidase activator	Produces ROS	[153, 154]
TfR1	Transferrin receptor protein 1	Mediates iron metabolism	[154, 155]
ACSL4	Acyl-CoA synthetase long-chain family member 4	Regulates the synthesis of fatty acyl CoA	[137, 154]
CARS	Cysteinyl-tRNA synthetase	Involved in the trans-sulfuration and synthesis of GSH	[156]
MAPK	Mitogen-activated protein kinase 1	Mediates cellular growth, survival, adhesion, and differentiation	[157–159]
NCOA4	Nuclear receptor coactivator 4	Regulates iron metabolism	[160–162]
15LO	15-lipoxygenases	Catalyzes the formation of pro-ferroptotic 15-OOH-AA(HpETE)	[163, 164]
SLC7A11	Cysteine/glutamate antiporter solute carrier family 7 member 11	Regulates the uptake of cysteine and the release of glutamate	[165–167]
PEBP1	Phosphatidylethanolamine-binding protein 1	Inhibits the Ras/MEK/ERK cascade	[168]
miR-182-5p	microRNA-182-5p	miR-182-5p correlates reversely with GPX4	[169]
miR-378a-3p	microRNA-378a-3p	miR-378a-3p correlates reversely with SLC7A11	[169]

The processes of AKI are very complicated. The extent to which ferroptosis participates in AKI still needs further research. The relationship between ferroptosis and iron hemostasis, ROS, and lipid peroxidation remained to be further addressed in AKI.

## The fundamental roles of organelles in ferroptosis

Organelles are important parts of cells that serve to maintain intracellular homeostasis. Recently, mitochondrial dysfunction, lysosome dysfunction, Golgi dysfunction, and ER dysfunction have been suggested to participate in the regulation of ferroptosis.

### Mitochondria

Previously, mitochondria were well-known energy stations for cellular activities and were closely associated with the process of PCD. The roles of mitochondria in ferroptosis have attracted great attention [66–68]. First, ferroptosis causes irreversible morphological changes in mitochondria [69]. Ferroptosis is characterized by a reduced density of the mitochondrial membrane, a smaller volume of mitochondria, decreased mitochondrial cristae, and a ruptured mitochondrial outer membrane (OMM). Second, mitochondria play predominant roles in iron utilization and anabolic and catabolic pathways [56, 70]. Increased iron uptake and mobilization lead to the deposition of intracellular iron in ferroptosis. Third, mitochondria regulate energetic metabolism in ferroptosis [21, 71]. Temporary activation of aerobic respiration is associated with oxidative injury to mitochondria in ferroptosis. Mitochondrial ROS play significant roles in ferroptosis. Fourth, mitochondria-lipid metabolism play critical roles in ferroptosis [72]. Mitochondria play vital roles in ferroptosis, where their morphology and function are irreversibly injured. The electron microscopy observation exhibited more swollen mitochondria in renal tissues of cisplatin-induced AKI animals than the normal controls, which could be alleviated by Fer-1 administration [73]. Accordingly, the HK-2 cells subjected to I/R were characterized by the shrinking of the shape, disorganization, and a reduction of the mitochondrial crista [55]. Targeting the mitochondrial-associated metabolic pathway might be a potential therapeutic strategy for ferroptosis-related diseases.

### Lysosomes

Lysosomes are well-known membrane-bound cell organelles that mediate the degradation of all kinds of biomolecules. Disordered lysosomes recruit undigested substances, which ultimately contribute to lysosomal storage dysfunction. Recently, researchers have pointed out that lysosomes play a significant role in the formation of ferroptosis [74–76]. First, lysosomes are the major storage location for iron [77]. The activity of lysosomes could affect intracellular iron provision by alleviating intracellular transferrin transportation or autophagic degradation of ferritin. Second, lysosomes are the main resource of cellular ROS in chemical-induced ferroptosis [69, 78]. The destabilized membrane of lysosomes leads to the release of large amounts of intracellular ROS, which subsequently contributes to cell death, including ferroptosis. Third, lysosomes are the main subcellular structure for the autophagic degradation of protein aggregates [79, 80]. Additionally, ferroptosis is often regarded as an autophagic cell death pathway. This was further confirmed by Gao, who found that autophagy inhibition greatly alleviated cysteine deprivation-induced ferroptosis [81]. Chen et al. suggested that legumain promotes ferroptosis in IRI-AKI. And the protective effects of legumain deficiency depend on its inhibition of lysosomal degradation [82]. However, direct evidence demonstrating ferroptosis as a consequence of autophagy is still lacking.

### Endoplasmic reticulum (ER)

As a subcellular organelle, the ER participates in the synthesis and exportation of proteins and lipids [10, 83]. Excess accumulation of unfolded proteins leads to ER stress. Moderate ER stress maintains the balance of intracellular homeostasis. Persistent ER stress contributes to apoptosis and cell death [10].

Recent studies have demonstrated that ER stress is involved in ferroptosis [84–86]. Zhang et al. found enhanced renal ER stress and the activation of ferroptosis in animal models of IRI-AKI [54]. The roles of ER stress in ferroptosis could be summarized as follows: first, ferroptosis inducers (erastin) trigger ferroptosis and ER stress together [87, 88]. Activated ER stress in turn inhibits ferroptosis and promotes drug resistance in a variety of tumors. Hence, the combined use of ferroptosis inducers and ER stress inhibitors in the treatment of cancer might be of great significance. Second, ER stress accelerates ferroptosis and worsens disease progression in some pathological conditions [89, 90]. Hence, the internal regulatory mechanism between ER stress and ferroptosis still needs further research. Third, ER stress is involved in the molecular crosstalk between ferroptosis and apoptosis [91]. The combined treatment of erastin or artesunate (ART) and tumor necrosis factor-related apoptosis-inducing ligand (TRAIL) can trigger apoptosis but not ferroptosis [91]. Erastin treatment alone does not lead to apoptosis. This result suggested that the relationship between ferroptosis and ER stress might be anergic or uncertain. ER stress might be the bridge between ferroptosis and apoptosis.

### Golgi stress

The participation of Golgi stress in ferroptosis has rarely been studied [92]. First, Golgi-dispersing agents, including BFA, AG1478/tyrphostin, AMF-26, and GCA, induce ferroptosis [92, 93]. Second, Golgi stress leads to a decreased intracellular glutathione pool, accumulation of lipid peroxides, and regulation of ferroptosis-associated signaling [92]. Third, iron chelators, antioxidants, overexpression of GPX4, and ACSL4 inhibition can prevent Golgi stress-induced ferroptosis [92]. Fourth, inhibitors of ferroptosis play beneficial effects on the function and morphology of the Golgi after Golgi stress inhibition administration [92].

Previously, studies on Golgi stress and ferroptosis mainly focused on cancer progression [94]. However, evidence in AKI is missing and still needs further research.

### The interplay between mitochondria, lysosomes, ER, and Golgi in ferroptosis

**Fig. 3:**
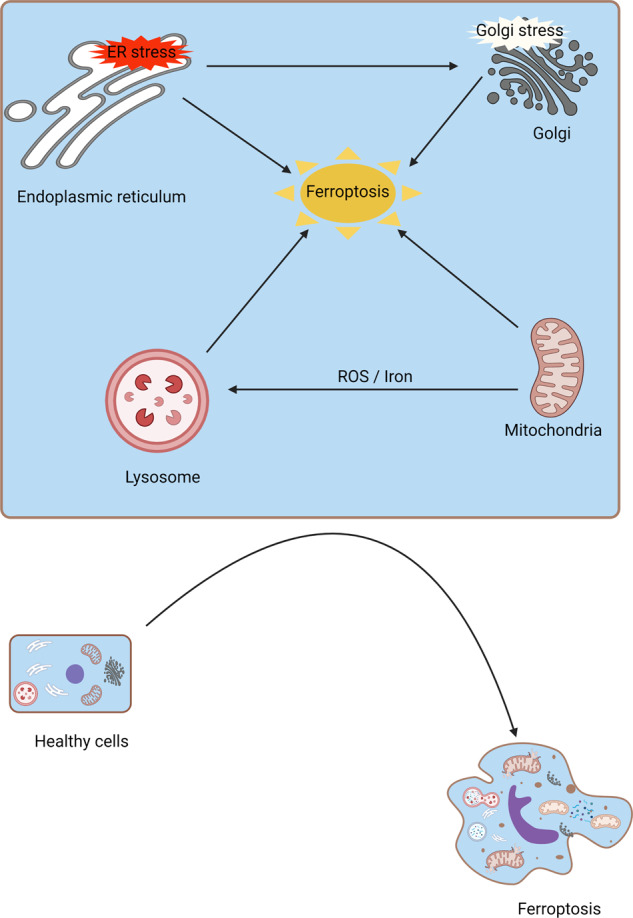
The interplay between mitochondria, lysosomes, the ER, and the Golgi in ferroptosis. Mitochondria and lysosomes together participate in iron metabolism, which induces ferroptosis. In addition, mitochondrial ROS can be taken up by lysosomes, ultimately leading to ferroptosis. ER stress can induce the function of the Golgi, which regulates ferroptosis.

Studies on organelle regulation have attracted great attention, especially in the field of ferroptosis [95, 96]. As we mentioned above, there is a close interplay between mitochondria, lysosomes, the ER, and the Golgi in ferroptosis (Fig. 3). First, mitochondria and lysosomes together participate in iron metabolism, which is the main mechanism of ferroptosis induction [77, 97]. Second, mitochondrial ROS can be taken up by lysosomes [69]. Excessive intracellular ROS lead to ferroptosis. Third, the capacity of the Golgi apparatus is modified according to ER stress [98]. However, the mechanisms of the organelle stress response are largely unclear. Investigations into the basic mechanisms of organelle regulation should receive much attention.

## Ferroptosis-related biological response in AKI

### Necroptosis

Necroptosis is a common feature in AKI. Cells undergoing necroptosis exhibit disrupted cell membranes, which is the major difference from necrosis. The RIPK3 and its substrate MLKL play critical roles in the formation of necroptosis [99–101]. In acute ischemic kidney injury, the expression of acyl-CoA synthetase long-chain family member 4 (ACSL4, a key enzyme in ferroptosis) is increased and is correlated with the severity of renal damage [82, 102]. Using CRISPR/Cas9 technology, Muller et al. found that knockout of *Acsl4* conferred protection from erastin- and RSL3-induced cell death in vitro [103]. Additionally, deletion of *MLKL* restrained susceptibility to necroptosis. Ferroptosis and necroptosis are alternatives in some cases, which means that resistance to one pathway sensitizes cells to death via the other pathway [103]. Diego et al. demonstrated that ferroptosis, but not necroptosis, is the major cause of cell death in folic acid-induced AKI [104]. Enhanced ferroptosis promotes the renal inflammatory response, which aggravates renal insufficiency. While, in other cases, ferroptosis and necroptosis act in a synergic or sequential fashion.

Collectively, one regulated necrosis pathway compensates for another when either ferroptosis or necroptosis is comprised.

### Autophagy

Autophagy is a dynamic intracellular degradation process that includes the formation and maturation of some membrane structures, such as autophagosomes, phagophores, and autolysosomes. Classical ferroptosis activators (such as erastin and RSL3) could increase autophagic flux in AKI [82, 105, 106]. Appropriate autophagy facilitates cell survival, while excessive autophagy might induce ferroptosis.

Ferritinophagy can regulate cellular iron through the autophagic degradation of ferritin [107]. Nuclear receptor coactivator 4 (NCOA4)-mediated ferritinophagy promotes iron accumulation in ferroptosis. In addition, RAB7A (a member of the RAS oncogene family)-mediated lipophagy, SQSTM1-mediated clockophagy and heat shock protein 90 (HSP90)-mediated chaperone-mediated autophagy (CMA) can induce ferroptosis by promoting lipid peroxidation.

Numerous studies have demonstrated that autophagy aggravates ferroptosis [108–110]. The mechanisms could be summarized as followed: First, autophagy generates lysosomal ROS. Second, autophagy leads to GSH depletion. Third, autophagy enhances lysosome cell death. Fourth, autophagy triggers lipid peroxidation. Fifth, autophagy recruits labile iron. While the crosstalk between autophagy and ferroptosis was complicated [111]. Recently, studies have also pointed out that both ferroptosis inducers and autophagy inducers facilitated the formation of autophagosomes via activation ER stress. Enhanced ER stress contributed to the activation of autophagy [109]. That’s to say, ferroptosis might also induce autophagy by ER stress. In addition, it had demonstrated selective autophagy or regulators of autophagic machinery in enhancing ferroptosis [107].

Collectively, autophagy is a lysosome-dependent cellular degradation process. Ferroptosis is an iron-dependent oxidative cell death pathway. In some conditions, ferroptosis has long been considered a form of autophagy-dependent cell death. Consequently, controlling the activity of autophagy during ferroptosis is of vital importance. However, the relationship between autophagy and ferroptosis is still controversial, which needs further research.

### Inflammation response

Emerging evidence has suggested the positive roles of ferroptosis in inflammation through immunogenicity [112, 113]. Ferroptosis in AKI can lead to the release of damage-associated molecular patterns (DAMPs) and alarmins, which further alert the innate immune system and shape the inflammatory response to cell death. Inhibitors of ferroptosis have anti-inflammatory effects in the process of AKI. The infiltration of neutrophils and the release of proinflammatory cytokines were alleviated by ferrostatin-1 (Fer-1, a ferroptosis inhibitor) in a mouse model of oxalate-induced AKI [114].

It should be noted that ferroptosis might be a double-edged sword. Therefore, it is of vital importance to determine the effects of ferroptosis on proinflammatory and anti-inflammatory responses.

### Interplay between ferroptosis, necroptosis, and inflammation in AKI

Ferroptosis is an early event that induces an inflammatory response in AKI [115], and necroptosis is a secondary wave that maintains and amplifies renal disorders. Ferroptosis occurs before the diagnosis of AKI. Hence, targeting ferroptosis could be beneficial for early prevention in AKI, while targeting necroptosis could be useful for further therapeutic intervention in AKI. While the process of renal IRI is very complex. It was proved that necroptosis and ferroptosis were synchronized renal tubular cell death in renal IRI [114]. Hence, the relationship between necroptosis and ferroptosis still needs further research.

## Ferroptosis in the AKI to chronic kidney disease (CKD) progression

**Fig. 4:**
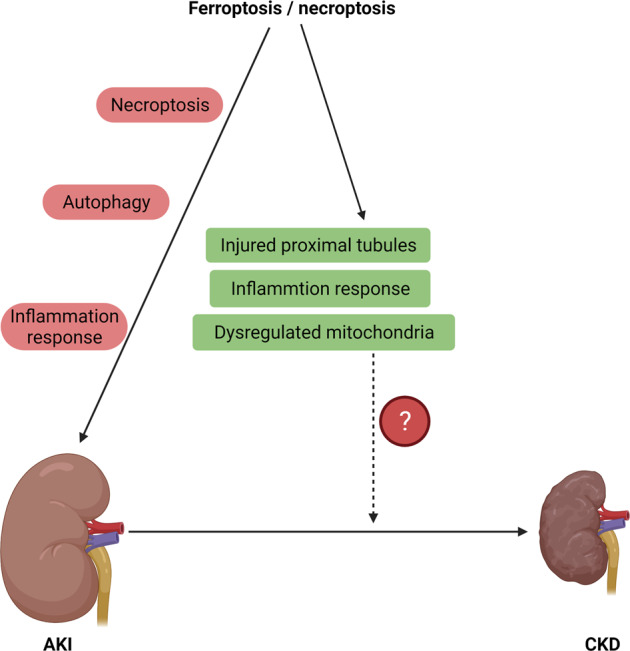
The roles of ferroptosis in AKI and the AKI to CKD transition. Ferroptosis induces necroptosis, autophagy, and inflammatory responses, which lead to the progression of AKI. In some cases, ferroptosis and necroptosis act in a synergic or sequential fashion. In addition, targeting ferroptosis can alleviate tubular injury, mitochondrial function, and the inflammatory response, which further contribute to the AKI to CKD transition. However, direct evidence of ferroptosis in the AKI to CKD transition is lacking.

A significant number of AKI survivors can progress to chronic kidney disease (CKD) and further to end-stage renal disease (ESRD). It was demonstrated that ~50% of hospital-associated AKI patients who survived AKI were newly diagnosed with CKD during a median follow-up period of 3.3 years [116]. Injured proximal tubules, dysregulated mitochondria, recruitment of immune cells, inflammatory responses, and heterogeneous backgrounds were described as the mechanisms of maladaptive repair after renal tubule damage [117]. Previous studies have suggested that targeting ferroptosis could prevent tubular injury, regulate the integrity of mitochondria, and alleviate inflammation via immune cells in rodent models of diabetic nephrology or AKI (Fig. 4) [118–121]. However, direct evidence about ferroptosis in the progression of AKI to CKD is lacking, which might be a hot point of research in the future.

## Ferroptosis in several rodent models of AKI

Emerging evidence has demonstrated the relationship between ferroptosis and AKI in vivo and in vitro. However, the specific regulatory mechanisms of ferroptosis in AKI are still largely unclear. Animal models for the investigation of ferroptosis are indispensable for further research [122].

### IRI-induced AKI

IRI, the most common reason for AKI, is characterized by a sudden pause in the blood supply to renal tissue and reoxygenation after blood flow is restored. AKI usually occurs due to clinical conditions, such as bleeding, dehydration, postoperative hypoperfusion, sepsis, and shock. Initially, apoptosis was thought to be the major kind of cell death in ischemic injury. In an in-depth study, necroptosis was regarded as the main cause of ischemic damage in the kidney and heart [123, 124]. Recent observations indicated that ferroptosis could be dominant in ischemic injury. In a clinical study, low levels of early intraoperative iron-binding proteins exhibited a damaged ability to rapidly process catalytic iron released during extracorporeal circulation, which further contributed to renal injury in AKI [125]. In IRI-induced mice, ferrostatin administration alleviated the function and organ damage [114]. In isolated tubule cells, ferroptosis inhibitors protected against hypoxic injury [126].

### Nephrotoxicity-induced AKI

Administration of nephrotoxic drugs is a common cause of renal injury and is widely applied for AKI research.

Folic acid (FA) triggers AKI in animals. A certain dose of FA might induce crystals in the renal lumen, while high doses of FA might further damage the tubular epithelium [127]. High levels of renal lipid peroxidation were observed in a mouse model of FA-AKI [104]. Pretreatment with Fer-1 alleviated the function and structure of the kidneys [104]. Interestingly, blocking necrotic apoptosis or apoptosis at the pharmacological or genetic level could not similarly reverse renal damage [104]. Thus, ferroptosis might be the major mechanism for regulatory necrosis in FA-AKI.

Cisplatin is a well-acknowledged antitumor agent. The feature of nephrotoxicity has attracted great concern [73, 128, 129]. Through experiments with a cisplatin-induced AKI model in vitro and in vivo, Baliga et al. suggested that cisplatin treatment led to increased expression of non transferrin-bound iron [130]. Additionally, deferoxamine (a chelating agent for removing excess iron) treatment significantly improved the functional and histological deterioration in cisplatin-induced AKI [131, 132].

Severe acute pancreatitis (SAP)-induced AKI is common in the clinic. AKI leads to drastic increases in mortality associated with SAP [11, 133]. The mechanism by which SAP contributes to AKI is still under investigation. It has been demonstrated that the production of inflammatory mediators and various cytokines and the accumulation of ROS are involved in the process of SAP-AKI. The SAP-AKI model was established by perfusion of sodium taurocholate (5%) into the biliopancreatic duct in rats. Ma et al. suggested the participation of ferroptosis in a rat model of SAP-AKI, and ferroptosis was associated with renal damage [134].

**Fig. 5:**
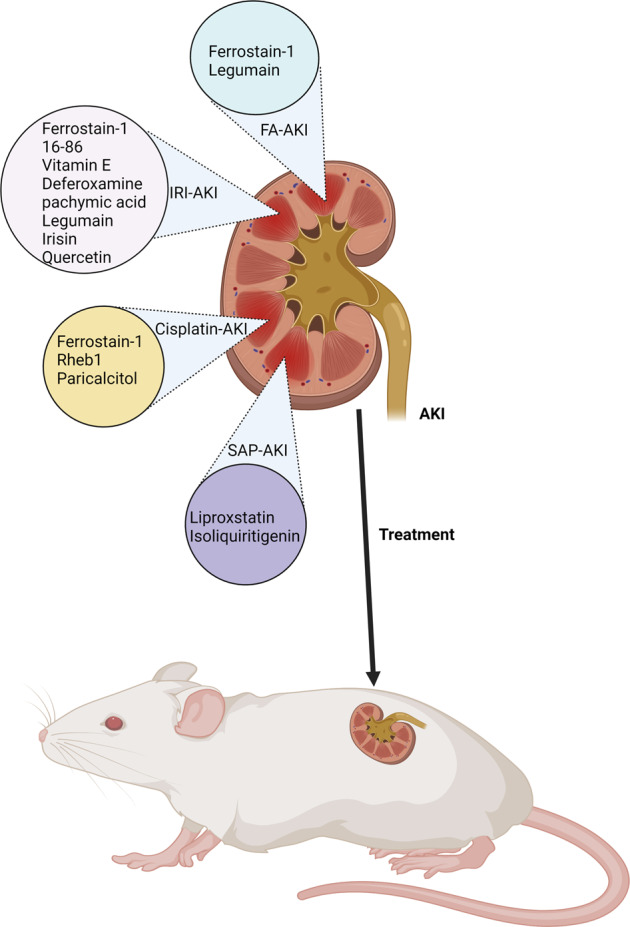
Ferroptosis-targeted treatment in AKI. In rodent models of AKI, pharmacological inhibitors of ferroptosis can be employed to alleviate ferroptosis in AKI.

Here, we briefly summarized the commonly used rodent models for the study of AKI (Fig. 5). Each model has its advantages and disadvantages. The IRI models seem to be the most popular and economic choices. Except for IRI, nephrotoxic models are suitable for the study of basic and common mechanisms of ferroptosis in AKI.

## Pharmacological advances regarding ferroptosis in AKI

From a therapeutic point of view, pharmacological inhibitors of ferroptosis can be employed to alleviate ferroptosis in AKI [135]. These include inhibitors of system Xc^−^, GPX4, iron chelators, lipophilic antioxidants, and liproxstatins, which inhibit lipid peroxidation and are summarized in Table 3. How much benefits could we obtain from ferroptosis-targeting strategies in AKI, and even in AKI to CKD still need more in-depth studies. More comprehensive research on ferroptosis in the field of AKI to CKD is encouraged to broaden our understanding and techniques against renal damage.

**Table 3:** Ferroptosis-targeted therapies in AKI.

Agents	Target	Diseases and models	References
Ferrostatin-1	Inhibits lipid peroxidation	IRI-AKI, cisplatin-AKI, FA-AKI	[104, 168, 170]
16-86	Inhibits lipid peroxidation	IRI-AKI	[114]
Vitamin E	Lipophilic antioxidant	IRI-AKI	[171]
Deferoxamine	Iron chelator	IRI-AKI	[172]
Rosiglitazone	Inhibits ACSL4	GPX4^−/−^ mice	[173]
Liproxstatin	Inhibits lipid peroxidation	GPX4^−/−^ mice, SAP-induced AKI	[50, 134]
Rheb1	Maintains mitochondrial homeostasis	Cisplatin-AKI	[174]
Pachymic acid	Activates NRF2 and upregulates GPX4, SCL7A11, and HO-1	IRI-AKI	[175]
Legumain	Facilitates chaperone-mediated autophagy of GPX4	IRI-AKI, FA-AKI	[82]
Paricalcitol	Upregulates GPX4	Cisplatin-AKI	[73]
Isoliquiritigenin	upregulates the system Xc^−^ and GPX4	SAP-AKI	[176]
Irisin	Upregulates GPX4	IRI-AKI	[54]
Quercetin	upregulates the system Xc^−^ and GPX4	IRI-AKI	[177]

Note: IRI-AKI ischemia and reperfusion injury-induced acute kidney injury, FA-AKI folic acid-induced acute kidney injury. Rheb1 Ras homolog enriched in brain.

## Conclusion

Ferroptosis is a newly discovered PCD that is involved in lipid peroxidation and iron accumulation. It is different from other forms of cell death, such as apoptosis, necrosis, and autophagy, in terms of its distinguishable morphology, biochemistry, and genetics. The current studies summarized the introduction, detection, and mechanism of ferroptosis. The levels of iron and lipid oxidases are the most important features for the measurement of ferroptosis. The mechanism of ferroptosis mainly involves four processes: GPX4, system Xc^−^, iron metabolism, ROS, and lipid peroxidation. In addition, ferroptosis can be regulated by several small molecules and genetic manipulation, weaving a puzzling and intricate relationship network.

The participation of ferroptosis in AKI has been identified. The roles and mechanism of ferroptosis in the progression of AKI and therapeutic strategies targeting ferroptosis in AKI have been discussed in our review. To better understand ferroptosis in AKI, some animal models have been established, such as IRI-AKI and nephrotoxicity-AKI. From a therapeutic point of view, pharmacological treatment targeting ferroptosis could be conducted to alleviate ferroptosis in AKI.

## Perspectives

The discovery of ferroptosis in AKI is still new, and associated research is greatly needed. Several issues should be addressed in future studies.

First, the current studies on the roles of ferroptosis in AKI came from in vivo and in vitro studies. More reliable animal models needed to be explored. Clinical studies are also encouraged.

Second, methods for the easy and reliable detection of ferroptosis are still lacking. Currently, the determination method is intrinsic, nonspecific, and time-consuming. Therefore, it is necessary to explore highly accurate and specific methods.

Third, current studies on ferroptosis have mainly focused on cancers. Mechanistic investigation of ferroptosis in AKI would have a good academic market, especially in the progression of AKI to CKD. In addition, ferroptosis participates in several renal diseases, such as diabetic nephrology and CKD.

Fourth, as a distinguishable PCD, ferroptosis occurs before the diagnosis of AKI. Early and precise detection of ferroptosis might be a novel biomarker for the diagnosis of AKI and might be useful in clinical applications.

Although the above obstacles do exist and should be addressed in future studies, we believe that focusing on ferroptosis is essential and promising. Some attention, as well as increasing hope, should be anticipated by clinicians as researchers perform large-scale studies regarding ferroptosis.

## Supplementary information

Reproducibility checklist

## Data Availability

The data used to support the findings of this study are available from the corresponding author upon request.
